# Applying the Plan-Do-Study-Act (PDSA) approach to a large pragmatic study involving safety net clinics

**DOI:** 10.1186/s12913-017-2364-3

**Published:** 2017-06-19

**Authors:** Jennifer Coury, Jennifer L. Schneider, Jennifer S. Rivelli, Amanda F. Petrik, Evelyn Seibel, Brieshon D’Agostini, Stephen H. Taplin, Beverly B. Green, Gloria D. Coronado

**Affiliations:** 1 Kaiser Permanente Center for Health Research, 3800 N. Interstate Ave, Portland, OR 97227 USA; 2 Lean HealthCare West, 315 SW 5th Avenue, Suite 900, Portland, OR 97204 USA; 3 Multnomah County Health Department, 426 SW Stark St, 8th Floor, Portland, OR 97204 USA; 4 Process of Care Research Branch, Behavioral Research Program, National Cancer Institute, Division of Cancer Control and Population Sciences, Rockville, MD USA; 5 Group Health Research Institute, 1730 Minor Avenue, Suite 1600, Seattle, WA 98101 USA

**Keywords:** Plan-Do-Study-Act, Colorectal cancer, Fecal immunochemical test, Process improvement

## Abstract

**Background:**

The Plan-Do-Study-Act (PDSA) cycle is a commonly used improvement process in health care settings, although its documented use in pragmatic clinical research is rare. A recent pragmatic clinical research study, called the Strategies and Opportunities to STOP Colon Cancer in Priority Populations (STOP CRC), used this process to optimize the research implementation of an automated colon cancer screening outreach program in intervention clinics. We describe the process of using this PDSA approach, the selection of PDSA topics by clinic leaders, and project leaders’ reactions to using PDSA in pragmatic research.

**Methods:**

STOP CRC is a cluster-randomized pragmatic study that aims to test the effectiveness of a direct-mail fecal immunochemical testing (FIT) program involving eight Federally Qualified Health Centers in Oregon and California. We and a practice improvement specialist trained in the PDSA process delivered structured presentations to leaders of these centers; the presentations addressed how to apply the PDSA process to improve implementation of a mailed outreach program offering colorectal cancer screening through FIT tests. Center leaders submitted PDSA plans and delivered reports via webinar at quarterly meetings of the project’s advisory board. Project staff conducted one-on-one, 45-min interviews with project leads from each health center to assess the reaction to and value of the PDSA process in supporting the implementation of STOP CRC.

**Results:**

Clinic-selected PDSA activities included refining the intervention staffing model, improving outreach materials, and changing workflow steps. Common benefits of using PDSA cycles in pragmatic research were that it provided a structure for staff to focus on improving the program and it allowed staff to test the change they wanted to see. A commonly reported challenge was measuring the success of the PDSA process with the available electronic medical record tools.

**Conclusion:**

Understanding how the PDSA process can be applied to pragmatic trials and the reaction of clinic staff to their use may help clinics integrate evidence-based interventions into their everyday care processes.

**Trial registration:**

Clinicaltrials.gov NCT01742065. Registered October 31, 2013.

## Background

The Plan-Do-Study-Act (PDSA) cycle is a commonly used improvement process in health care settings that might have untapped potential for pragmatic research. A PDSA activity uses small tests of change to optimize a process. A pragmatic clinical trial called the Strategies and Opportunities to STOP Colon Cancer in Priority Populations (STOP CRC) recently used this process to optimize the implementation of a cancer screening outreach program. Unlike traditional trials, pragmatic clinical trials, occur in real-world settings where everyday care happens; as such, they require extensive collaboration between researchers and the staff of a health care system [1–6]. This paper describes how our study used PDSA in pragmatic research to assist in improving implementation of the planned intervention (a mailed outreach program); however, this technique could have broader applications in a wide range of quality improvement activities and other types of pragmatic trials.

While PDSA cycles are commonly used in clinical care, few clinical research trials have documented its use for implementation. Standard clinical trials emphasize internal rather than external validity, using highly controlled environments and selected populations. In contrast, pragmatic studies are generally embedded in care delivery environments. Few research-based interventions can be used ‘off the shelf’; adaptations are often needed to accommodate unique aspects of a setting or population. Since the PDSA cycle is a clinical improvement process, it is often familiar to clinical staff. Therefore, the PDSA cycle may prove useful in adapting and implementing research-based interventions, particularly where its incorporation into every-day care is a central question.

Several investigators have reported on clinical change interventions using PDSA cycles [7–9]. Vetter used PDSA cycles to pilot test a clinical decision support system (CDSS) among nurses to improve diagnostic accuracy. Both Stevens et al. [9] and Hendricks [8] successfully used the PDSA framework to implement a substantial clinical practice change. The former used PDSA cycles to gradually roll out a chronic care model for residents while the Hendricks study improved breast cancer patients’ adherence to oral antiemetics through enhanced patient education and better documentation. Malloy et al. [10] reduced wait times for patients and improved patient satisfaction, notably changing clinical processes in a way that made staff invested in the changes and interested in further improvement at 3 months follow-up.

We describe how we embedded the PDSA approach into our pragmatic research study, STOP CRC, which involved 26 Federally Qualified Health Center (FQHC) clinics. STOP CRC is testing an electronic health record (EHR)-embedded program to identify patients due for colorectal screening and to mail fecal immunohistochemical test (FIT) kits to them. Colorectal cancer screening rates are lower in FQHC clinics than in national data (2015 data: 38% vs. 63%), [11, 12] and direct-mail FIT testing programs are demonstrated to improve rates of CRC screening in this setting [13–17].

This paper presents PDSA topics chosen by clinic staff and the results. Our study team worked with clinic staff to identify the question they wanted to ask and to determine what data they needed to collect and by whom (Plan), carry out the change or activity and collect the data (Do), study the data collected (Study), and identify next steps or further PDSA cycles (Act). We also conducted qualitative interviews with project leads from the participating centers to highlight the reactions of clinical staff to incorporating the PDSA program in the study.

## Methods

### Participating sites

The 26 clinics participating in the STOP CRC study are operated by eight health centers in Oregon and Washington. All are safety-net clinics that are affiliated with OCHIN [formerly the Oregon Community Health Information Network]. OCHIN is a health care innovation company specializing in technology for safety-net clinic health care services. The step-wise intervention (involving a mailed introductory letter, mailed FIT kit, and mailed reminder letter) requires clinic staff to identify eligible patients by using EHR reporting tools developed by OCHIN for this study.

**Fig. 1:**
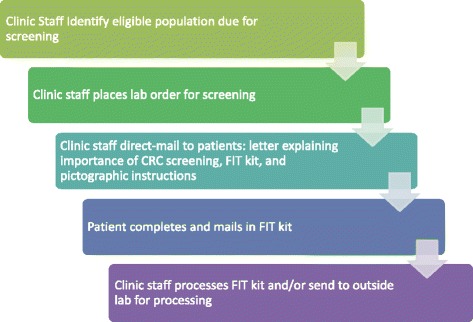
STOP CRC clinical workflow

The STOP CRC intervention workload was handled at the health center level, sometimes by a centralized department and other times by clinical teams. Once the eligible population was identified, clinic staff placed a lab order, mailed a letter to patients that explained the importance of testing for colorectal cancer (CRC), and provided the patients with FIT test kits with pictographic instructions. Clinic staff tracked returned FIT kits, which were either processed on-site or at an outside laboratory. Finally, clinic staff managed patient follow-up, which included reminding patients who did not return kits, recording test results (if the lab interface was not automated), notifying patients of results, and referring patients to colonoscopy services when indicated (Fig. 1).

### Description of PDSA work with clinics

As a standard quality-improvement process, the PDSA cycle helps introduce a new program into a complex environment, such as primary care. An improvement process, for example, may identify the need for a workflow that can improve efficiency (e.g., calling patients with invalid addresses) or training (e.g., best practices for recording historical colonoscopies). This process can also identify additional intervention components to improve effectiveness or reach (e.g., clinic posters that show how to do the test). While PDSA activities are often conducted as multiple small cycles to implement a change, we asked intervention clinic staff to conduct and report on only a single PDSA cycle as part of the pragmatic trial. Because of the heterogeneity of our sites, we assessed each clinic’s quality improvement infrastructure and experiences with the PDSA process before we asked those sites to conduct an activity.

**Fig. 2:**
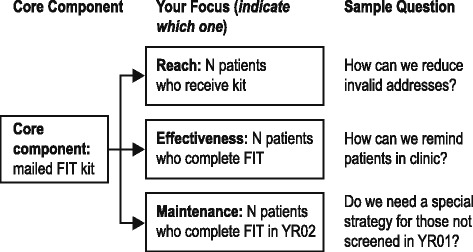
PDSA Framework for STOP CRC

The PDSA itself was conducted by clinic staff at their intervention site. In January and early February of 2015, STOP CRC project team members (GC, SR, AP, JR) met with clinic leadership (e.g., the project lead, medical director) to discuss deliverables. These meetings included 1 or 2 individuals from Lean Healthcare West, who provided orientation and brief training. During this 1.5-h meeting, clinics were given examples of PDSA activities developed by the STOP CRC team. Lean Healthcare West also provided PDSA job aids, PDSA plan templates, and a report template. After this meeting, the clinic was sent a presentation template for the problem statement, a PDSA framework, a plan for data collection tasks (to document who, what, where, when, and how data will be collected), an outcomes table (to collect outcome and data source and how they would be gathered), and a plan of next steps (Fig 2).

Each clinic’s PDSA plan was due February 2015 and the activity was expected to be completed between March and May 2015. Clinic staff measured their own PDSA results and reported back to the research team in June 2015. PDSA results were shared with the STOP CRC study at advisory board meetings in June 2015 and September 2015. A meeting with one clinic was delayed to January 2016 due to staff turnover at that clinic.

### Qualitative data collection procedures

Working with the project leads from each health center, we identified and recruited via email key staff involved in implementing STOP CRC for an in-depth telephone interview. Interviews were conducted by study staff (JLS, JSR) approximately when health centers would have completed their PDSA cycle. An interview guide, developed by the research team, covered topics related to barriers and facilitators of project implementation and explored why the key staff chose their topic, their perspectives of PDSA outcomes, and the benefits and challenges of engaging in the PDSA process. All interview procedures and materials were reviewed by the Institutional Review Board at Kaiser Permanente Northwest and were qualified for a waiver of signed consent. The telephone interviews lasted about 45 to 60 min and occurred between June and July 2015.

We used qualitative content analysis [18–21], to develop and apply a coding dictionary to the verbatim telephone transcripts [22, 23]. We focused primarily on codes and transcript content pertaining to the PDSA experience. Transcripts were coded by research staff trained in qualitative analysis methods (JLS, JSR) and entered into Atlas.ti version 5.0, a qualitative analysis software program used to electronically code and manage data and to generate reports of coded text for ongoing thematic analysis [20, 22, 23]. Coded text was subsequently reviewed by the research team through an iterative process that resulted in refined themes.

## Results

Table 1 presents the aim of each clinic’s PDSA and what it would test for the STOP CRC study. The aim statement identifies a topic area for improvement.

**Table 1:** PDSA Cycle aims and high-level plans as submitted by health clinics

Health Center	PDSA Aim Statement	Initial Plan
Correct Workflow and Staffing		
Health Center 1	Create standardized process for CRC screening.	Test staffing models for mailing FIT kits. By June 1, 2015, have a standard workflow to increase percentage of patients screened for CRC.
Health Center 2	Develop standard work for printing letters and mailing kits that can be sustained by support staff within teams. (~100 mailings per month).	Test scenarios for using alternative staffing models (like the front desk staff) and temporary staff to prepare and mail FIT kits.
Health Center 3	Compare return rates from kits distributed in-clinic vs. kits mailed, and shorten the look-back period for mailed kits from 1 year to 3 months.	Pilot-test pre-visit planning to improve capture of CRC screening data in the medical record.
Increase Return Rate		
Health Center 4	Improve the rate of FIT kit returns.	Test the mailing of the introductory letter with and without FIT and assess results for patients enrolled or not enrolled in the patient portal.
Health Center 5	Determine whether a second reminder via phone call will increase the rate of FIT kit returns.	Test phone reminders.
Health Center 6	Improve the rate of FIT kit returns.	Test the use of metered return mailing versus drop off at the clinic.
Health Center 7	Improve the rate of FIT kit returns.	Test the mailing of FIT 1–2 weeks prior to scheduled clinic visit.
Increase Accuracy of FIT Collection		
Health Center 8	Resolve the issue that many completed FIT kits cannot be processed because the patient omitted the date(s) of collection on the kit label.	Improve materials to prompt patients to write the date of collection on the kit label.

*CRC* colorectal cancer, *FIT* fecal immunochemical test

### Initial clinic plans for STOP CRC PDSA cycles

Clinics identified areas in need of improvement for which they could implement a change and measure the results. Clinic plans for the PDSA cycles fell into three general groups with similar reasons for choosing their PDSA.

1. **Address staffing needs and improve workflow of the intervention**. Two centers tested staffing models that might increase the number of letters and FIT kits mailed to patients. A third center wanted to verify CRC information from patients’ medical records to confirm eligibility, thereby improving workflow.
2. **Increase rate of FIT kits returned by patients**. Three centers tested ways of mailing reminders to patients to increase the return rate of the mailed kits. An additional center assessed whether phone reminders would increase the return rate.
3. **Increase usability of FIT kits returned**. One center focused on altering materials to improve the rate of patients returning the kit with the dates of collection.

### PDSA outcomes

The following sections provide representative examples of clinic PDSA activities and findings from completed PDSA cycles.

### Correct workflow and staffing: Health center 2

Health Center 2 exemplifies a PDSA designed to clarify the roles of staffing and improve workflow. Clinic staff examined whether the number of letters and FIT kits sent to patients could be boosted by using staff at the front desk rather than lab technicians or clinical staff. Local university students working at Health Center 2 educated staff about the importance of CRC screening and use of FIT testing, created a patient education tool, and improved workflow.

“We chose to see how we were going to transition this work to the clinical staff… because at the time of starting the PDSA we had this backlog of work, and I [as project lead] had no way of knowing how we were going to be able to give this to the clinical team, which is where I thought it belonged. I also knew that I had some nursing students coming and colorectal cancer screening was something they wanted to work on. And so I thought this was a great opportunity to figure that out. So that’s why I chose it.” – Clinic Project Lead

The evaluation involved comparing the number of introductory letters mailed, FIT kits mailed, and FIT kits completed from September 2014 through February 2015 (before the staffing change) and (March 2015 – April 2015) (post-change). The new staffing system resulted in about 3.0, 2.8, and 1.5 times as many mailings of introductory letters, mailings of FIT kits, and FIT kits returned, respectively.

### Increase return rate: Health center 4

Health Center 4 had a lower than expected rate of return of their FIT kits. This clinic decided to test whether mailing the introduction letter and FIT kit together rather than separately would improve the rate of returned FIT kits. Health Center 4 mailed their FIT kits based on patients’ birth month because their population was too large to mail all the kits in 1 month. The STOP CRC project began mailing kits at Health Center 4 in June 2014; the PDSA method was implemented for the December 2014 mailing.

“The time that it takes to batch the letters, mail the [introduction] letters, and then batch the kits and mail the kits [second mailing] is one reason [we chose our PDSA topic] - that would just cut down on one step. And then another reason is we didn’t really know if the patients weren’t returning the kits because they’d never opened the intro letter, or vice versa. And so if we mailed it all together then they don’t have to find the first piece of mail that we sent them that reminds them why they’re getting a second piece of mail.” – Operations Manager

In conducting their PDSA process, Health Center 4 learned more about the functionality and limitations of their reporting tool, EPIC’s Reporting Workbench (which was the EHR registry tool used to implement mailed interventions). To conduct the PDSA activity, Health Center 4 used the Reporting Workbench list of kits mailed and matched it to an EHR FIT report to obtain the lab result date. This report also showed if the order was canceled (expired) or was still outstanding (see PDSA data in Table 2). Prior to implementing the PDSA workflow change (combined introduction letter and FIT kit), the average clinics’ return rate was 19.8%; after the PDSA workflow change, the average clinics’ return rate was 23.2%.

**Table 2:** Health center 4 return rates before and after PDSA change

	Pre-PDSA^a^		Post-PDSA^b^	
Site	N	% Returned	N	% Returned
Clinic 1	568	18.5	421	23.0
Clinic 2	144	25.0	311	23.5
Total	712	19.8	732	23.2

^a^Eligible patients with birthdays between June 2014 and November 2014, before a combined introduction letter and FIT kit

^b^Eligible patients with birthdays between December 2014 and February 2015, after workflow change of separately mailed introduction letter and FIT kit

### Increase return rate: Health center 5

Health Center 5 also implemented a PDSA activity to increase FIT kit return rates, but with a reminder phone call to patients.

“We were wondering if we could increase the rates of return of kits for resulting if we were to make an additional reminder phone call to people that had still not returned a kit following their reminder letter. After reviewing a report showing the remaining unreturned kits, the panel manager called patients on the list.” – Project Lead

Using the department’s standard process, 282 out of 403 FIT kits that were mailed (84.5%) were not returned. Clinic staff called each of these patients once and left a voicemail message if the patient was not reached directly. After staff had made 215 phone calls, ten patients called back and requested an additional kit; only one person returned a completed test. The extra reminder call did not substantially increase the number of completed tests in this cycle.

### Increase accuracy: Health center 8

Health Center 8 realized it needed to change its intervention materials to improve the quality of sample collection. Their FIT test included a card for sampling and required that patients write the appropriate collection date for each of two stool samples. Laboratory staff at Health Center 8 were concerned about the number of samples that were unusable because patients were omitting the collection dates on the test card.

“We had a very large percentage of returned samples coming back without the collection date on the label — patients didn’t understand that they needed to put the collection date on the label. So when those samples came back, even if they were viable samples, if they didn’t have a collection date we had to toss them. So we had to figure out a way to teach patients the importance of that and make sure that it was done.” – STOP CRC Project Lead

**Fig. 3:**
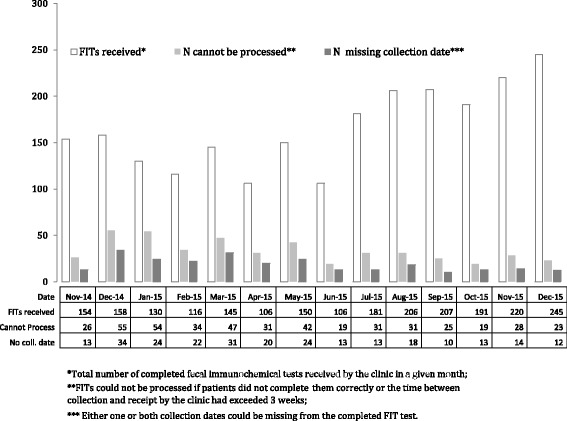
Findings from Health Center 8 PDSA that Addressed Completed but Invalid FIT kits

To address this problem, Health Center 8 staff highlighted in yellow the sentence in the introduction letter that instructed patients to write the collection date for each collected sample. They also developed a new FIT kit insert that included a graphical image of where to write the collection dates. The new workflow procedures began in June 2015. They tracked the monthly number of kits that were returned improperly completed and the number with omitted collection dates during two 7-month time periods (November 2014 through May 2015 and June 2015 through December 2015). They found that the average number of test cards with missing collection dates dropped from 24.0 to 13.3 and the average number of overall samples that were improperly collected dropped from 41.3 to 25.1 (Fig. 3).

### Qualitative interview findings

We completed telephone interviews with all eight health centers representing 22 participants in a range of roles (including project lead, operations or quality improvement manager, EMR specialist, and medical assistant) working on the STOP CRC project and their PDSA activities.

All interview participants found their PDSA activity for STOP CRC beneficial. Primarily, participants found it helped them focus on the planning and organizing necessary to implement tasks pertaining to workflow, staffing, and resources for the program.

“There were still some unknowns and so it focused me in writing out what our problems actually were… so having it all down in one document really helped focus the project and [we] know what to work on next.” – Operations manager

Additionally, participants’ STOP CRC PDSA activities tested their assumptions, such as whether a reminder call would increase the return rate of FIT kits. Furthermore, executing the activity identified future steps, such as additional PDSA activities or data needs, to help each center decide whether and how to maintain the STOP CRC program.

“The PDSA itself was valuable in that it told us that making [a change] does not necessarily provide a significant return… [Our] next PDSA is probably going to be figuring out how to roll out the program to the first thousand people. ” – Project Lead

“PDSAs are always valuable… it would be really interesting to dig a little deeper into the demographic data to really narrow in on something [regarding return rate].” – Operations manager

Six of the eight health centers described the PDSA program as having a broader value beyond implementing STOP CRC and improving CRC screening rates. For those centers, the PDSA activity elucidated how the organization could engage in population-based outreach for other screening services. It also educated staff on EMR tools that support systematic outreach efforts, such as consistent use of and documentation within Epic’s Health Maintenance and Reporting Workbench. For the health center with the least experience using the PDSA cycles, the activity provided an opportunity to be trained in how to standardize its use.

“It helped us look at ways to do patient outreach for all of our other preventive care, and being able to use reporting workbench and generate letters to send those reminders… ” – Operations manager

“But the [PDSA] process itself, we kind of do that organically already without calling it a PDSA. So now it’s nice to have a form and a template that we can work by so that we can get feedback… and come up with questions like what about if we did this or who’s going to do that. So it’s good to have that template to work from.” – Quality Improvement manager

While the health centers saw benefits from engaging in the STOP CRC PDSA activity, the full PDSA ramifications were not apparent for any of the clinics at the time of the post-activity interview. For half of the centers, accessing data to gather the PDSA results and assess improvement outcomes was not easy or efficient. For example, some PDSA programs relied on monthly research project reports, which was too infrequent for assessing and refining the results in a timely way. For other health centers, accessing data using their clinical reporting systems was difficult and created time-consuming work-arounds. Moreover, because FITs take time to complete, return, and process, the impact of a given PDSA on FIT return rates took months to measure. The long measurement time had the added effect of making it difficult for clinics to isolate the precise cause of the response rate changes.

“…because of the nature of this project, I don’t get data on how we’re doing frequently enough to be able to make quick adjustments… So that is a problem that I’ve got, I need the data more regularly like weekly updates…” – Project Lead

“We’ve had a difficult time measuring the success of our PDSA because of the reports, or lack thereof, that we have available to use to see the total number sent versus the total number returned, so we’ve gotten creative with some things… the reports we have available in Reporting Workbench aren’t exportable… so we’ve transitioned to using some reports out of another reporting tool but it’s quite a tedious task to get that data too and it requires doing several steps, each one prone to error” – Operations manager

An important issue for three of the centers was the delay in starting the PDSA cycle due to staffing changes and the need to obtain approval from compliance leaders regarding some aspect of the PDSA program. The project lead of one center described challenges in designing and reporting on a single test of change (as outlined by STOP CRC), which differed from the iterative manner PDSAs are typically executed.

“We use PDSAs a lot here, and I’m used to really quick PDSA cycles where we try something and next week we see what went well or didn’t go so well… So the PDSA I am used to we would get really rapid feedback on how we’re doing so that we can adjust, so that has been something to get used to for this.” – Project Lead

### Post-PDSA planned changes

Each health center was asked to submit the changes they were planning (or not planning) to make in response to their PDSA findings. We report on the plans for the four health centers highlighted above.

Clinic staff at Health Center 2, whose PDSA activity involved staffing changes, continued to revise their clinical processes and optimize their use of EMR tools. They worked with OCHIN to print address labels (instead of printing addresses on the letters themselves) and began ordering kits in bulk (i.e., placing many lab orders at a time).

Health Center 4 planned to continue the combined mailing of the introduction letter with the FIT kit. Health Center 4 discussed alternative distribution methods and decided to spread the direct mailed program to all their clinics as the best way to reach all of their patients due for screening. Health Center 4 also quickly conducted additional PDSA cycles. Their second cycle involved a second reminder letter and their third involved replacing the second reminder letter with a phone call. A fourth activity, tested at a small site in April 2016, shifted their process to allow patients to either drop off FIT kits or mail them back to the clinic.

Health Center 8 staff had good results from altering the mailed materials so that FIT kit users could understand where to write the collection date. The staff planned to continue to monitor the volume and reasons for unprocessed specimens. They kept the additional steps in preparing their kits for mailing.

In contrast, Health Center 5 learned that calling patients to remind them to return the FIT kit took too much time and effort. They are now considering instead an additional mailed postcard with contact information to remind patients to return their kits.

## Discussion

We asked clinics implementing a fairly complex intervention to use a PDSA cycle to evaluate and refine the intervention soon after implementation. While past research has used a PDSA process to implement research interventions, our study used a PDSA cycle to enable clinics to improve or refine the program. The results revealed that many clinics chose to work on questions of better implementation (i.e., staffing and workflow) and gave us insight into the implementation challenges faced by clinic staff.

The PDSA activities uncovered implementation issues of which a research team would not typically be aware. For example, Health Center 8 sought to increase the proportion of fecal tests that included a collection date so that the outside lab could process the results, and so they focused their activity on modifying patient materials that highlighted this instruction to patients. The PDSA activity illuminated an issue that had hindered the effectiveness of the intervention, diminished patient satisfaction with testing by requiring them to retake the test, and created extra work for the clinic team. While solving this issue was critical to us as a research team it was also fundamental to clinical care. This site was motivated to increase their screening rate and determined that they needed tests that were appropriately completed. It was in both of our interests to increase the proportion of tests that were appropriately completed and processed; implementing a PDSA cycle led to that result.

The PDSA approach in several clinics focused on defining or refining the staffing model in busy community practices, which surprised us. Those approaches revealed that clinic staff did not anticipate the intervention’s complexity and the staffing needed to implement the program nor the complexity of using new EHR reporting tools. Also, patient management at the population level was relatively new for the clinics at the time of the intervention. For example, they were not necessarily staffed with panel managers working on prevention across patient groups, and the timeline for implementation of the intervention shifted due to staff turnover at a few clinics. Therefore, when we launched the PDSA improvement process 4 to 6 months “after” the intervention, several clinics were still trying to implement the intervention by mailing letters and FIT kits.

### Use of PDSA program in pragmatic trials

PDSA cycles could be a novel method for dealing with the complexity of implementation. At the heart of the PDSA method is the ability to break things down and focus on making small, measurable changes [24]. Our study was a little different in that we asked clinics to select and report on a single change that might lead to subsequent iterative changes. The usual PDSA change is very small and the results are gathered and evaluated very quickly. Having each clinic select a single focus led to clinics choosing a slightly broader PDSA scope, which could have made their results harder to measure. Furthermore, several clinics went on to conduct multiple PDSA cycles, each in rapid succession. In these clinics, the PDSA was useful in refining the initial intervention to improve outcomes and clinic workflow.

Using PDSA cycles in a pragmatic research study might help address the trialability of an intervention. Trialability is the ability to experiment with an innovation for either a limited time or one piece at a time [25]. This characteristic of new interventions relates to their likelihood of adoption and is included in the Consolidated Framework for Implementation Research. A high level of trialiability may accelerate the adoption of a given intervention by making it testable within a clinic environment prior to full implementation. Because new innovations require investing time, energy and resources, organizations prefer innovations that can be tried before being fully implemented [26]. Another study found that organizations that base their changes on results are more likely to adopt an innovation if it is perceived as open to experimentation [27]. Although trialability is not consistently important for adoption of every innovation, it may be especially important for high-risk, expensive, complex, or obtrusive innovations [28].

PDSA cycles may be particularly valuable in implementing or adapting research-based interventions that leverage EHRs. In our experience, health record-enabled interventions require in-depth understanding of existing clinical processes and the alignment between tool functionality and clinical workflows. Changing a multi-step process can have consequences that lead to smaller steps in incremental process improvement.

Clinical staff had positive reactions to the use of PDSA cycles in the STOP CRC project. As part of pragmatic research, the PDSA process helped engage the clinics more fully in their research. The PDSA process may have served a role in inviting clinic staff to collaborate in a shared goal of how best to optimize the implementation of the direct-mail program in a busy clinical practice. Staff involved in the process felt it was useful in helping to identify and solve their problems. Nonetheless, staff also desired improved electronic systems for tracking the outcomes.

The PDSA process is widely used and familiar to many involved in clinical care. As such, it offered familiarity and our training provided more in-depth exposure to how it could be used. Simple exposure to this process could improve other aspects of clinics’ care. More important, it allows clinic staff to own the study process [24, 29].

Our use of PDSAs in pragmatic research raises some critical questions. Is the best time to implement a PDSA process during the initial intervention (as in prior research) or months into the process (as we did)? Given our emphasis on implementation (vs. effectiveness) the PDSA approach was well suited for our study but may not be appropriate for all. The program seems to work best for identifying implementation challenges and optimizing the implementation of evidence-based intervention into practice. If the research question emphasizes efficacy or effectiveness over implementation, then variation in how a program is implemented may threaten its validity. Given our emphasis on implementation and reliance on clinic staff to deliver their programs, it made sense for clinic staff to tailor the program’s implementation to their unique context. The PDSA appears to have been a useful tool for accomplishing that.

There were several limitations to our study. First, the standard PDSA cycle involves multiple iterations but we asked clinics to just do one cycle for the study, which may have affected what project each clinic chose. Second, a few clinics experienced issues with collecting data to support their PDSA work. Third, due to staff changes, the timing of our PDSA cycles at some of the clinics happened before the intervention was fully implemented, which likely shifted the focus of the improvement cycle. Regardless of these limitations, our results demonstrate that the PDSA is a viable technique to use in pragmatic research.

## Conclusions

In summary, we reported on the novel and effective use of the PDSA cycles in pragmatic research. Its use can illuminate how an intervention is actually being implemented by health systems. Further, using PDSA in pragmatic research can help uncover implementation challenges and may enable clinics to integrate a research-based intervention into everyday care processes.

## Abbreviations

PDSA: Plan-Do-Study-Act
STOP CRC: Strategies and Opportunities to STOP Colon Cancer in Priority Populations
CRC: Colorectal cancer
EHR: Electronic health record
FIT: Fecal immunohistochemical test
